# Demodulation of Chaos Phase Modulation Spread Spectrum Signals Using Machine Learning Methods and Its Evaluation for Underwater Acoustic Communication

**DOI:** 10.3390/s18124217

**Published:** 2018-12-01

**Authors:** Chao Li, Franck Marzani, Fan Yang

**Affiliations:** 1 State Key Laboratory of Acoustics, Institute of Acoustics, Chinese Academy of Sciences, Beijing 100190, China; 2 University of Chinese Academy of Sciences, Beijing 100190, China; 3 LE2I EA7508, Université Bourgogne Franche-Comté, 21078 Dijon, France; Franck.Marzani@u-bourgogne.fr (F.M.); fanyang@u-bourgogne.fr (F.Y.)

**Keywords:** underwater acoustic communication, direct sequence spread spectrum, chaos phase modulation sequence, partial least square regression, machine learning

## Abstract

The chaos phase modulation sequences consist of complex sequences with a constant envelope, which has recently been used for direct-sequence spread spectrum underwater acoustic communication. It is considered an ideal spreading code for its benefits in terms of large code resource quantity, nice correlation characteristics and high security. However, demodulating this underwater communication signal is a challenging job due to complex underwater environments. This paper addresses this problem as a target classification task and conceives a machine learning-based demodulation scheme. The proposed solution is implemented and optimized on a multi-core center processing unit (CPU) platform, then evaluated with replay simulation datasets. In the experiments, time variation, multi-path effect, propagation loss and random noise were considered as distortions. According to the results, compared to the reference algorithms, our method has greater reliability with better temporal efficiency performance.

## 1. Introduction

The underwater acoustic communication has always been a crucial research topic [1,2,3,4,5,6]. In some special applications, high-performance communication is required, resulting that the system must satisfy the constraints of reliability, security and efficiency simultaneously. The direct-sequence spread spectrum (DSSS) communication is one of the most effective potential solutions to the problem of confidential underwater acoustic communication. It spreads the frequency spectrum of the carrier wave with a spreading code sequence, so the modulated signal is hard to be detected by a third party within the underwater noise, possessing greater concealment [7,8].

**Figure 1:**
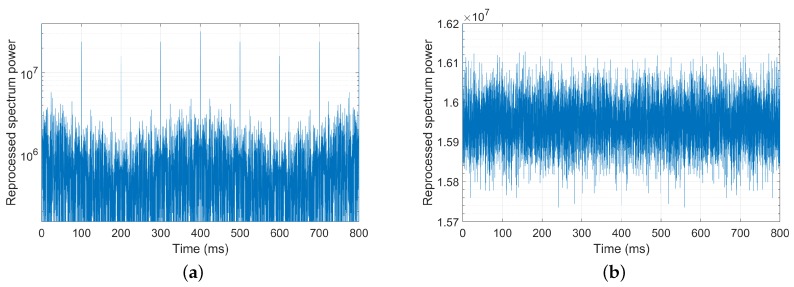
Period estimations of m- and chaos sequence spread spectrum signals with power spectrum density (PSD) reprocessing [15]. (**a**) m-sequence; (**b**) Chaos sequence.

Conventional DSSS technique is used to modulating the carrier wave with pseudo-noise (PN) sequences, such as m-, Gold and Kasami sequences [9,10,11]. However, these PN sequences can only provide finite keyings and limited code resources, (e.g., binary phase-shift modulation keying, quadrature amplitude modulation, frequency shift keying), so the transmitted DSSS signals usually possess binary-value and periodic characters. Estimating the modulation parameters from the signals of this type is not very hard even with the blind methods [12,13,14,15]. For example, Figure 1a plots the power spectrum density reprocessing $S_{rep}$ of the m-sequence spread spectrum signal computed as follows:

(1) $$S_{rep}\left(g\right)={\left|F\left[S\left(f\right)\right]\right|}^{2}={\left|\int_{-\infty }^{+\infty }S\left(f\right)e^{-i2\pi gf}df\right|}^{2}$$

where S is the power spectrum of the received signal, and F refers to the Fourier Transform. Due to the periodicity of m-sequence, regular spikes occur at the integer multiple of the sequence period along the time axis, the period of the transmitted signals can be, therefore, accurately estimated by measuring the interval width between two spikes [15].

Over the recent twenty years, chaos theory has been incorporated into the conceptions of PN sequences for the benefits of broad band, irregular complexity and orthogonality [16,17,18]. Usually, chaos (deterministic chaos) refers to irregular, unpredictable behavior in deterministic, dissipative, and nonlinear dynamical systems [19]. Within DSSS, it can be considered as a kind of disorder sensitive to initial conditions, allowing to generate a large number of orthogonal chaos sequences by setting different initial values. This method is helpful to overcome the binary-value and periodic problems of old code sequences. As the simulation results shown in Figure 1b, adopting chaos sequence significantly increases the difficulties of the parameter retrieving. Up till now, a series of ingenious chaos maps have been proposed (e.g., logistic [20], tent [21], cubic [22], Chebyshev [23], Bernoulli [24], multi-segment piecewise linear maps [25]), considerably improving communication security.

Despite of multiple advantages, adopting chaos spread spectrum into real-life underwater acoustic communication is far from easy. The de-spreading and demodulation of chaos spread spectrum signals at the receiver is challenged by the signal distortions caused by time and space variations of underwater environments, reverberation, multi-path effect, low signal-to-noise ratio, etc. [26]. Matched filter is one of the most widely used demodulators in underwater acoustic communication [27]:

(2) $$s_{out}\left(t\right)=\int_{-\infty }^{+\infty }H\left(f\right)S\left(f\right)e^{j2\pi ft}df$$

where $s_{out}$ is the instantaneous output of the filter, H is its transfer function and S is the spectrum density function of the given signal s. When the channel impairment is only the white gaussian noise, according to the Cauchy–Bunyakovsky–Schwarz inequality, the optimal matched filter is, therefore, $H\left(f\right)=\alpha S^{*}\left(f\right)e^{-2\pi ft_{o}}$, where α and $t_{o}$ are, respectively, a constant value and the time delay, implying that its impulse response is actually the replica of s. The matched filters obviously do not have the ability to suppress the non-Gaussian-distributing noise and channel multi-path effects. In underwater communications, it is, therefore, usually combined with the equalization techniques or passive-phase conjugation method to mitigate the distortions form the channels of this type [7,28,29,30]. Stojanovic et al. successfully realized the deep-water 204-km 660-bps, shallow-water 89-km 1000-bps, and shallow-water 3.7-km 10-kbps underwater communications with equalization optimizations, respectively [28]. It is claimed that a passive-phase conjugation-based communication method achieved an average bit error ratio (BER) of $10^{-6}$ at SNR=−5 dB with simulations [7], and a successful communication with the distance of 900 km in a deep-water experiment [31].

Yet, unlike common acoustic signal detections which distinguish the interested signal from the ambient noise, chaos spread spectrum signal demodulation is a recognition task that is much more complicated. It has to find out the right class of the received symbol from a large candidate set, necessitating the target classification capacity via feature analysis. In our opinions, today’s underwater sound communication techniques can be further improved from the following two aspects:

- High-performance symbol classification method. Though the conventional matched filters are able to enhance the symbol energy over noise by providing a processing gain with maximal SNRs, all of the components of the symbol sequences or vectors are unintentionally considered to contribute equally to the model precision without feature analysis, resulting in values of the key components that are diluted by the others. Hence, there are still some opportunities to further improve the accuracy performance of the demodulators through feature analysis.
- Noise analysis. Submarine noise is one of the main interferences with the quality of underwater communication. They come from a variety of sources, such as marine life, sea quakes, rain, artificial constructions, etc. The ambient noise of transducers are highly random, and the analysis of their statistical distribution feature helps potentially to improve the reliability of underwater communication modalities.

For the issues mentioned above, we focus our work on the investigation of machine learning-based chaos spread spectrum signal demodulation techniques. It is motivated by the fact that machine learning methods handle the classification problem by making data-driven predictions or decisions through building a statistical model from sample inputs. It provides a nice solution to statistically analyze the data features from the given sample sets. Because this procedure is automatic, it is especially appropriate to establish the physical models which are hard to be described using a precise analytic function. Within underwater communications, the received signals are distorted from the transmitted signals via the underwater sound channels with random noise interferences, so it can be considered that they contain all of the necessary factors of the propagation model. Learning directly from the original data with a proper approach may help to build a precise model to benefit underwater communication techniques from signal and environment feature analysis.

This paper is devoted to the underwater acoustic communication scheme. A new proposed chaos phase modulation method for underwater communication [32] is used to modulate the communication symbols. The chaos phase modulation method first arose in the 1990s [33,34] and was then used in radar systems [35,36] and high-performance multi-channel secure underwater communications [32]. With chaos phase modulation techniques, a synchronized copy of the chaotic symbol signal is generated at the receiver side and the demodulation is performed by exploiting this replica in different methods to recover the transmitted data, which is known as a coherent communication system [37]. Compared to the nonherent systems, such as differential chaos shift keying, chaos phase modulation does not have the problem of low data transmission rate, in which half of bit energy is used to transmit the reference signal [38,39]. Because it modulates the phases of the carrier wave with chaos sequence, which can provide a huge number of modulation keyings and code resources, it can also overcome the problem of periodicity and binary values caused by conventional PN sequences. Furthermore, the signals of this type have nice orthogonality, providing high distinguishebility between symbols, which can potentially facilitate the demodulation task at the receiver end.

This paper innovatively processes the demodulation of chaos phase modulation signals as a target recognition problem, so it can be solved using either matched filter or machine learning methods. For the purpose of low bit error rate, a partial least square (PLS) regression-based demodulation framework is conceived. PLS algorithm is initially a standard tool for processing a wide spectrum of chemical data problems. Its success in chemometrics resulted in a lot of applications in other scientific areas including bioinformatics [40], food research [41], medicine [42], pharmacology [43], social sciences [44], etc. Recently, this regression method has been successfully used in hyperspectral image recognition applications in the biometric field [45,46], which effectively improve the test accuracy by modeling the relations between training and prediction matrices. The use of PLS regression in chaos phase modulation communications allows us to weigh the received discrete signal through statistical analyses, further raising the accuracy of the system.

The proposed demodulation scheme is implemented with a multi-core center processing unit (CPU). The evaluation experiments of this paper are conducted using simulation datasets, within which the time variation, multi-path effect, propagation loss and random noise are considered as distortions. In order to obtain unbiased results, two reference algorithms are implemented with different optimization forms. The results demonstrate that the proposed method achieves the best accuracy performance with high temporal efficiency within the experimental protocol of this paper.

The remainder of this paper is organized as follows: Section 2 describes the chaos phase modulation scheme; Section 3 and Section 4 present the proposed demodulation method and its parallel implementation, respectively; Section 5 analyzes the evaluation experiment results; finally, a conclusion is given in Section 6.

## 2. Message Signal Modulation

This paper modulates the phases of the communication symbols directly on the complex exponential function [32]:

(3) $$p_{l}=exp\left\{j2\pi k_{l}\right\}$$

where $k_{l}\in \left[-0.5,0.5\right]$ is the *l*-the element of chaos sequence. We can see that chaos phase modulation sequences are complex sequences with randomly-distributed phases, and their envelopes are constant.

We use the multi-segment piecewise linear mapping [25,47] to generate the desired chaos sequence k. It is an iteration procedure that can be programmatically expressed as follows:

(1) Initialize the first element of the sequence $k_{0}$ with a random value, known as “seed” in computer science;
(2) If $k_{l}>1/2$, repeat the computation $k_{l}:=k_{l}-\left|2\xi k_{l}\right|$ with ξ∈[0,1] until the constraint is satisfied, otherwise, go to the next step;
(3) If $k_{l}<-1/2$, repeat the computation $k_{l}:=k_{l}+\left|2\xi k_{l}\right|$ until the constraint is satisfied, otherwise, go to the next step;
(4) Compute the next element $k_{l+1}$:
  (4) $$k_{l+1}=\left\{\begin{array}{cc} 2\left(N-\xi \right)k_{l}+\left(N-0.5\right)\; & -1/2⩽k_{l}<\left(1-N\right)/\left(2N\right)\\ 2\left(N-\xi \right)k_{l}+\left(N-1.5\right) & \left(1-N\right)/\left(2N\right)⩽k_{l}<\left(2-N\right)/\left(2N\right)\\ & \vdots \\ 2\left(N-\xi \right)k_{l}+\left(-N+0.5\right) & \left(N-1\right)/\left(2N\right)⩽k_{l}<1/2 \end{array}\right.$$
(5) If l=L−1, task completed, otherwise go back to the second step.

Steps (2)–(4) above correct iteratively $k_{l}$ to keep it in the given interval with the help of a step coefficient ξ∈[0,1]. *L* is the length of sequence k. Step (4) segments the interval [−0.5, 0.5] into 2N pieces ($N\in N^{+}$). Now we can create the chaos spreading spectrum symbol u(t):

(5) $$u\left(t\right)=\sum_{l=0}^{L-1}d\left(t\right)\times g\left(t,l,p_{l}\right)$$

with

(6) $$\begin{array}{c} d\left(t\right)=A\cdot \frac{e^{j2\pi f_{0}t}+e^{-j2\pi f_{0}t}}{2} \end{array}$$

(7) $$\begin{array}{c} g\left(t,l,p_{l}\right)=\left\{\begin{array}{cc} p_{l} & \mathrm{if}\;t\in \left(\frac{T\cdot l}{L},\frac{T\cdot (l+1)}{L}\right]\\ 0 & \mathrm{otherwise} \end{array}\right. \end{array}$$

where d(t) is the carrier wave, *A* is amplitude, $f_{0}$ is the frequency of carrier wave, *g* is a shaping function, $T=T_{c}\times L$ is the time length of symbol and $T_{c}$ is the time length of chip. In this work, cosine signal is used as the carrier wave.

We can write the fourier transform of u(t) as follows:

(8) $$\begin{array}{cc} F[u(t)] & =\sum_{l=0}^{L-1}\int_{-\infty }^{+\infty }d\left(t\right)\times g\left(t,l,p_{l}\right)\times e^{-i2\pi ft}dt \end{array}$$

(9) $$\begin{array}{c} =\sum_{l=0}^{L-1}\int_{\frac{T\cdot l}{L}}^{\frac{T\cdot (l+1)}{L}}d\left(t\right)\times e^{i2\pi (k_{l}-ft)}dt \end{array}$$

The equation above implies that chaos phase modulation associates the frequency spectrum of u(t) with the chaos sequence k. According to the chaos theory, chaos sequences are sensitive to the initial conditions, even if they are approximately equal. Consequently, correlators will have a low output unless they are highly synchronized with the inputs. That allows to create a large number of highly orthogonal symbol candidates by changing the seeds.

**Figure 2:**
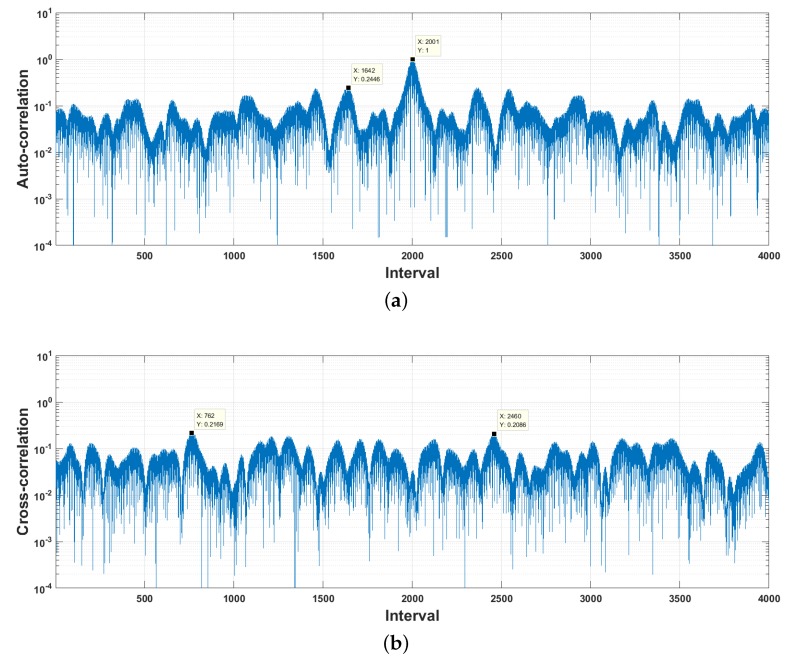
Correlation of chaos phase modulation signals. (**a**) Autocorrelation; (**b**) Cross-correlation.

Figure 2a plots the normalized autocorrelation of a chaos phase modulation signal measured by using a classical matched filter (see Equation (2)). The correlation values of main and side lobes are marked. We can see clearly that their ratio is around 4.1×, whereas, as shown in Figure 2b, it is hard to distinguish the main and side lobes on the cross-correlation curves, and the maximum cross-correlation coefficient is only 0.217, demonstrating that chaos phase modulation signals have nice orthogonality.

For the purpose of textual communication, we map the 8-bit ASCII table to chaos phase modulation signals. Each ASCII code is represented by using a symbol with certain time length, performing a symbol candidate set for message edition at the transmitter and signal demodulation at the receiver.

## 3. Signal Demodulation

We consider the demodulation problem as a symbol recognition one. This section describes the proposed demodulation scheme at the receiver.

### 3.1. Architecture of Demodulator

**Figure 3:**
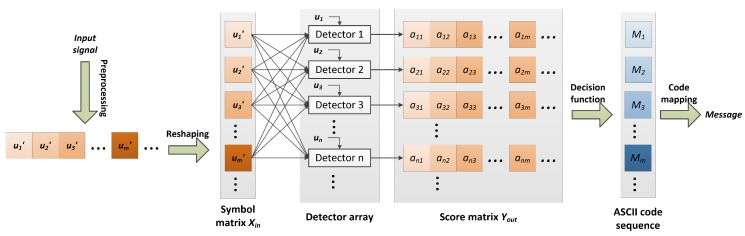
Overall flowchart of demodulation.

Figure 3 shows the demodulation flowchart of this paper. The input signal is first resampled and pre-processed via a band pass filter, then reshaped into the symbol matrix $X_{in}$. The rows of $X_{in}$ correspond to the received symbols $u_{m}^{\prime }$, and the columns to the sampling points. Thirdly, $u_{m}^{\prime }$ are associated to the units of a detector array, respectively. The units of detector array are essentially binary classifiers, which score the similarity degree between $u_{m}^{\prime }$ and the symbol candidate $u_{n}$. The output of the detector array is a score matrix denoted using $Y_{out}$, whose rows correspond to the symbol candidates and the columns to the received symbols. The element of the score matrix $a_{nm}$ represents the similarity between $u_{n}$ and $u_{m}^{\prime }$. Finally, the transmitted message is recovered through a decision function and ASCII code mapping. This paper supposes that a higher score means better similarity; the decision function, therefore, returns the indices of the maxima of the columns of $Y_{out}$:

(10) $$M_{m}=max\left\{a_{m}^{T}\right\}$$

where $a_{m}^{T}=\left[a_{1m},a_{2m},\ldots ,a_{nm},\ldots \right]$.

### 3.2. PLS-Based Detection Method

**Figure 4:**
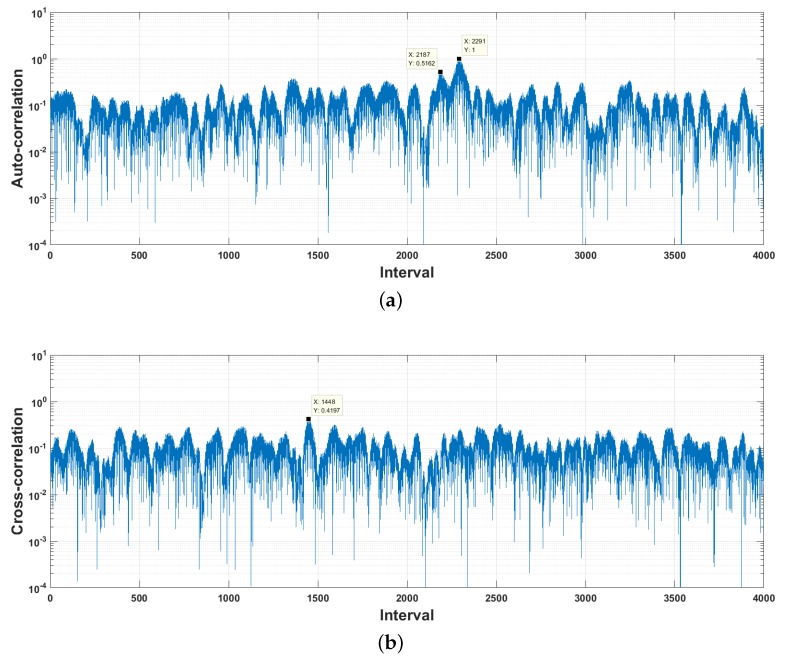
Correlation between the original chaos phase modulation signal and its underwater sound channel output at SNR=−10 dB. (**a**) Autocorrelation; (**b**) Cross-correlation.

Signal detection plays an important role in the low-BER acoustic communications. One of the most frequently used acoustic signal detection methods is the matched filter (see Equation (2)). The underwater sound channels and ambient noise usually distort the message signals seriously, resulting in marked degradation in signal detection performance. Figure 4a plots the autocorrelation between the original chaos phase modulation signal and its observation at the receiver simulated by using the sound channel response estimated from the trail data. We can see that the main-to-side lobe ratio goes from 4.1× (see Figure 2) down to lower than 1.94×. Whereas, the maximum cross-correlation increases from 0.217 up to 0.42, see Figure 4b.

The simulations above demonstrate that the distinguishability of the received chaos signals is reduced significantly during the underwater propagation. For the purpose of high accuracy performance, we use a partial-least square (PLS) regression-based classifier as the detector array in Figure 3.

PLS is a wide class of methods for modeling relations between sets of observed variables by means of latent variables. It comprises regression and classification tasks, as well as dimension reduction techniques and modeling tools. Projection of the observed data to its latent structure by means of PLS was developed by Herman Wold and coworkers [48,49,50]. The underlying assumption of all PLS methods is that the observed data is generated by a system or process which is driven by a small number of latent (not directly observed or measured) variables. Its goal is to maximize the covariance between the two parts of a paired dataset even though those two parts are in different spaces. This method is essentially a machine learning one, which necessitates the training and testing processes, simultaneously.

#### 3.2.1. Training Process of PLS Regression

Let x be a random example for training and y its response. Either x or y is zero-mean column vectors. In order to assess their relation with covariance, we project them onto two separate directions specified by unit vectors $w_{x}$ and $w_{y}$ in order to obtain two random variables $w_{x}^{T}x$ and $w_{y}^{T}y$. Now we present two matrices X and Y whose *i*-th rows are the feature vectors of corresponding examples $x_{i}$ and $y_{i}$. According to the nonlinear iterative PLS algorithm, PLS searches for the directions $w_{x}$ and $w_{y}$ such that [45,51]

(11) $$\max_{w_{x},w_{y}:||w_{x}\left|\right|=\left|\right|w_{y}\left|\right|=1}C\left(w_{x},w_{y}\right)=w_{x}^{T}C_{xy}w_{y}=\frac{1}{m}w_{x}^{T}X^{T}Yw_{y}$$

where $C_{xy}=\frac{1}{m}X^{T}Y$ is the covariance matrix of X and Y, and *m* is the example number. The direction that solve the maximal covariance optimization are the first singular vectors $w_{x}=U_{1}$ and $w_{y}=V_{1}$ of the singular value decomposition of $C_{xy}$:

(12) $$C_{xy}=U\Sigma V^{T}$$

where the value of the covariance is given by the corresponding singular value $\sigma_{1}$.

In this paper, more than one projection direction are wanted. To do this, the same strategy of data projecting is applied by deflation. This creates the new data matrix

(13) $$X^{\prime }=X\left(I-w_{x}w_{x}^{T}\right)$$

Let $P_{j}$ be $\frac{X_{j}^{T}X_{j}U_{j}}{X_{j}^{T}U_{j}^{T}U_{j}X_{j}}$ with j=1,2,…,k, where *k* is the projection direction number, the deflation of $X=X_{1}$ is obtained as follows:

(14) $$X_{2}=X_{1}\left(I-U_{1}P_{1}^{T}\right),\;\mathrm{with}\;X_{1}=X$$

Considering a test point with the feature vector φ(x), we define $\tilde{\varphi }\left(x\right)$ as the feature vector needed for the regression, whose columns are $\varphi_{j}{\left(x\right)}^{T}U_{j}$. The new feature vector can be expressed as follows:

(15) $$\tilde{\varphi }{\left(x\right)}^{T}=\varphi {\left(x\right)}^{T}\tilde{U}{\left(P^{T}\tilde{U}\right)}^{-1}$$

where $\tilde{U}=\left[U_{1},U_{2},U_{3},\ldots ,U_{j}\right]$.

Now, we can start to compute the regression coefficients vector W, which performs the regression of the variables Y in terms of $X\tilde{U}$. We seek a coefficient matrix B that solves the optimization

(16) $$\min_{B}||X\tilde{U}B-Y{\left|\right|}^{2}=\min_{B}\langle X\tilde{U}B-Y,X\tilde{U}B-Y\rangle$$

The final regression coefficients W are given by $\tilde{U}B$. We seek the minimum by computing the gradient with respect to B and setting it to zero. The overall regression coefficients can be computed as follows:

(17) $$W=\tilde{U}{\left(P^{T}\tilde{U}\right)}^{-1}C^{T}$$

where C is the matrix with columns $c_{j}=\frac{Y^{T}X_{j}U_{j}}{U_{j}X_{j}^{T}X_{j}U_{j}}$.

The PLS regression algorithm is shown in Algorithm 1. The repeat loop computes the first singular value by the iterative method. We can train the PLS model by assigning the training matrix $X_{train}$ and its responses $Y_{train}^{T}$ to X and Y, respectively. The element $Y_{train}\left(n,m\right)$ of $Y_{train}$ is 1 if the *m*-th received symbol $u_{m}^{\prime }$ is matched with the *n*-th symbol candidate $u_{n}$ and 0 otherwise. For a training matrix having 4 symbol candidates and two received symbols per candidate, $Y_{train}$ is as follows:

(18) $$Y_{train}=\left(\begin{array}{cccccccc} 1 & 1 & 0 & 0 & 0 & 0 & 0 & 0\\ 0 & 0 & 1 & 1 & 0 & 0 & 0 & 0\\ 0 & 0 & 0 & 0 & 1 & 1 & 0 & 0\\ 0 & 0 & 0 & 0 & 0 & 0 & 1 & 1 \end{array}\right)$$

**Algorithm 1** Pseudocode of PLS Regression Algorithm.**Input:** training matrix X, response variables Y, projection direction number *k* **Output:** regression coefficients W   1:centering the data  2:**for**j=1,2,…,k**do**  3:      $U_{j}\leftarrow$ first column of $X_{j}^{T}Y$  4:      $U_{j}\leftarrow U_{j}/\left|\right|U_{j}\left|\right|$  5:      **repeat**  6:            $\;U_{j}\leftarrow X_{j}^{T}YY^{T}X_{j}U_{j}$  7:            $\;U_{j}\leftarrow U_{j}/\left|\right|U_{j}\left|\right|$  8:      **until** convergence  9:      $P_{j}\leftarrow X_{j}^{T}X_{j}U_{j}/\left(U_{j}^{T}X_{j}^{T}X_{j}U_{j}\right)$10:      $c_{j}\leftarrow Y^{T}X_{j}U_{j}/\left(U_{j}^{T}X_{j}^{T}X_{j}U_{j}\right)$11:      $X_{j+1}\leftarrow X_{j}\left(I-U_{j}P_{j}^{T}\right)$12:**end for**13:$W\leftarrow \tilde{U}{\left(P^{T}\tilde{U}\right)}^{-1}C^{T}$

#### 3.2.2. Demodulation with PLS Regression

The symbol matrix to be demodulated $X_{in}$ is formed of the normalized received symbol ${\bar{u^{\prime }}}_{m}$:

(19) $${\bar{u^{\prime }}}_{m}=\frac{u_{m}^{\prime }-\mu }{\sigma }$$

where ${\bar{u^{\prime }}}_{m}$ is the normalized received symbol sequence and is stored as the rows of $X_{in}$, μ and σ are the mean and standard deviation of the training matrix $X_{train}$. We associate $X_{in}$ to the well-trained PLS model to compute the score matrix $Y_{out}$:

(20) $$Y_{out}^{T}=X_{in}\times W$$

The similarity score $Y_{out}\left(n,m\right)$ of $u_{m}^{\prime }$ related to the *n*-th symbol candidate $u_{n}$ is expected to be maximal if matched, otherwise 0. The index of the maximum of the score vector of $u_{m}^{\prime }$ (stored as the *m*-th column of $Y_{out}$) is considered as the estimated class of this received symbol.

## 4. Implementation and Optimization

The pseudocode of the demodulation algorithm of this paper is shown in Algorithm 2. In order to facilitate the description, the decision function in Figure 3 is inlined into the top function. After program initialization, the input signal is first filtered via an FIR filter for denoising, then segmented and reshaped into the form of the symbol matrix $X_{in}$ through the loop in Line 3. Thirdly, the PLS regression function PlsModel() is invoked. It returns the score matrix $Y_{out}$. Fourthly, findmax() function searches for the column maxima $Y_{out}$, and return their indices, performing an index vector ind. Finally, ind is assigned to the message generation function MesGen(). The desired message string is generated by mapping the indices to the characters in the ASCII table.

**Algorithm 2** Pseudocode of the Proposed Demodulation Algorithm.**Input:** input signal s, frequency band $f_{b}$, normalization coefficients μ and σ, regression coefficients W, sampling rate $f_{s}$, symbol time length *T*, ASCII table ascii **Output:** message str   1:initialization  2:$s^{\prime }\leftarrow fir\left(s,f_{b}\right)$  3:**for**i=1,2,…,m,…**do**  4:      $\bar{u_{i}^{\prime }}\leftarrow \left[s^{\prime }\left(\left(\left(i-1\right)\times f_{s}\times T+1\right):\left(i\times f_{s}\times T\right)\right)-\mu \right]/\sigma$  5:      $X_{in}\left(i,:\right)\leftarrow \bar{u_{i}^{\prime }}$  6:**end for**  7:$Y_{out}\leftarrow PlsModel\left(X_{in},W\right)$  8:**for** the *i*-th column of $Y_{out}$, i=1,2,…,m,… **do**  9:      $\mathrm{ind}\left(i\right)\leftarrow findmax(Y_{out}\left(i\right))$10:      str(i)←MesGen(ind(i),ascii)11:**end for**

According to Algorithm 2, the proposed demodulation algorithm is lightweight and possesses high parallelism. From the view point of programming, the loop in Line 3 reshapes the input signal sequence $s^{\prime }$ without any computational operations. PlsModel() function is essentially a matrix multiplier. The loop of Line 8 is an ideal independent loop, whose iterations can be pipelined or parallelized completely without initiation interval.

**Figure 5:**
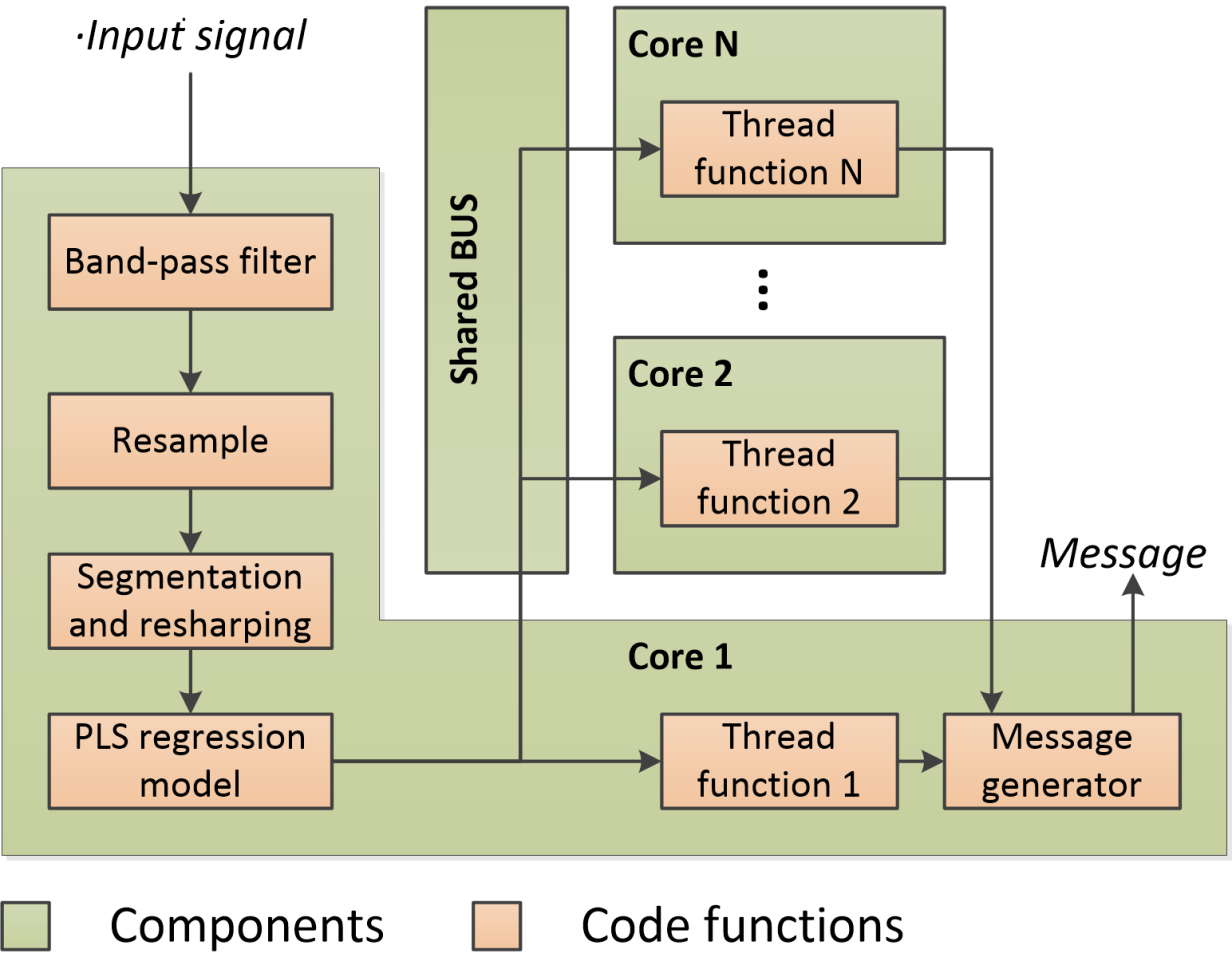
Architecture of the optimized demodulation implementation.

Hence, we optimized the original code for a multi-core CPU platform. Figure 5 shows the overall architecture of the optimized demodulation implementation. The CPU cores are interconnected with a shared bus. The first core of CPU masters the preprocessing, resampling, segmentation, reshaping tasks and PLS model. When these computations are finished, the score vectors stored in $Y_{out}$ are distributed to the other cores via a shared bus for ASCII mapping. The mapping task consists of findmax() and MesGen() functions. We parallelize them with the single instruction multiple data (SIMD) approach, which enables us to apply the same description code (thread function) to process different data in parallel. The thread functions formed of findmax() and MesGen() functions are distributed to the different CPU cores which share the memories and data bus. Since the data or operations are independent, all of the threads are able to execute simultaneously so that the running speed is accelerated.

## 5. Experiments and Evaluations

The proposed method is evaluated with the simulation experiments. In order to obtain an unbiased result, all the used datasets are simulated with the data measured and recorded in the sea trail. The user-controlled parameters of the algorithm are optimized experimentally. The accurate and computational performance of the proposed method are evaluated by comparison with the two reference algorithms.

### 5.1. Dataset

The experiments of this paper are conducted by using simulation data, which are generated via the replay simulation method presented in [52]:

(21) $$s\left(t\right)=\int_{\tau =0}^{T_{h}}h\left(t,\tau \right)\cdot s_{o}\left(t-\tau \right)d\tau +s_{n}\left(t\right)$$

where $s_{o}\left(t\right)$ is the transmitted signal, s is the received signal, h is the measured underwater sound channel response, $T_{h}$ is the tap number of the channel response and $s_{n}$ is the ambient noise.

**Figure 6:**
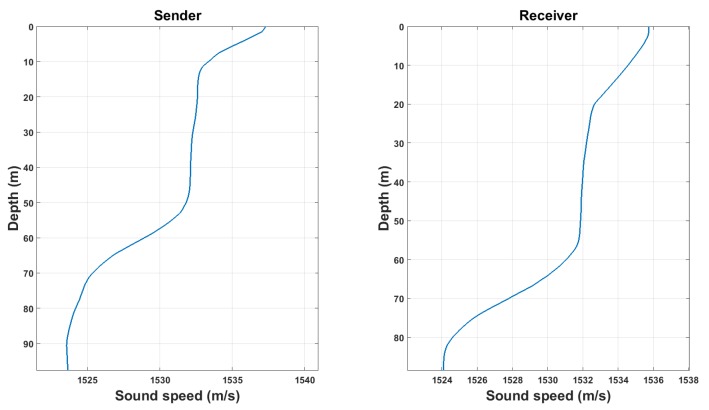
Smoothed sound velocity profiles at the sender (**left**) and receiver (**right**).

Equation (21) allows to simulate time variation, multi-path effect, propagation loss and random noise occurred during the underwater acoustic propagations by specifying the channel response h and ambient noise $s_{n}$. The real-life underwater noise is used as the ambient noise in this work. The sound channel response is estimated from a set of experimental data recorded by the Institute of Acoustics of Chinese Academy of Sciences in the South China Sea. Figure 6 plots the smoothed sound velocity profiles at the sending and receiving sites. The acoustic signal was transmitted from a fixed source to a fixed receiver array. Table 1 displays the parameters of the experimental environment and channel estimation. The time coherence of the experimental environment is evaluated by measuring the normalized correlation of two 10-s chaotic signals with a time interval of 5 min. The result value of 0.82 demonstrates that the underwater sound channels are highly correlated during the measurement time.

**Figure 7:**
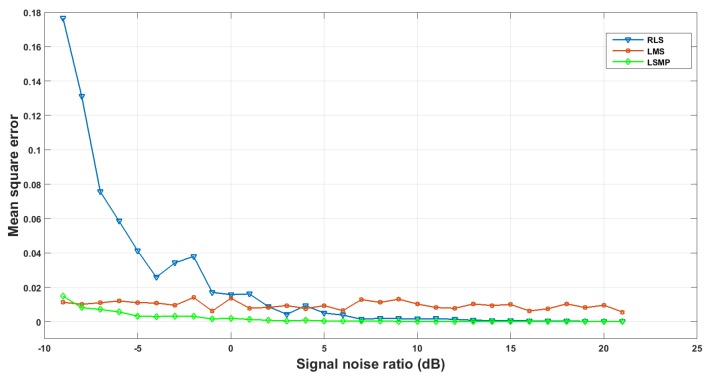
Accuracy comparison of channel estimation methods.

In order to obtain an accurate ocean channel, three frequently used channel estimation methods are evaluated for selection, including least mean square (LMS) [53], recursive least squares (RLS) [54] and least squares matching pursuit (LSMP) [55] algorithms. Figure 7 plots the mean square error (MSE) between the original channel response and the ones measured with the three methods over the signal-to-noise ratio. It is obtained with the simulated sparse channel response and spreading frequency signal. Finally, we selected LSMP for its high reliability and accuracy.

**Figure 8:**
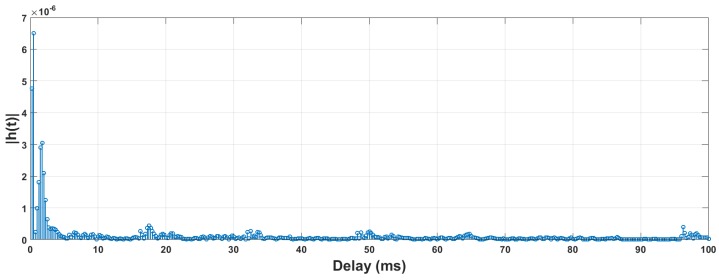
An example of channel response measured using LSMP algorithm.

Figure 8 plots a channel response measured using the LSMP algorithm. It can be seen that the histogram possesses multiple peak points, representing the different multi-path arrivals [55]. We do not normalize it in order to keep the attenuation information. The normalized correlation coefficient between the original probe signal and the signals simulated with Equation (21) is 0.79.

Now, we can start to generate the desired dataset with Equation (21). Given an 8-bit ASCII code, there are 256 symbol candidates u; for each one, we generate six sessions of simulated signals $u^{\prime }$. The dataset, therefore, contains 256 candidates ×6 sessions =1536 simulated symbols.

### 5.2. Parameter Configuration

The subject of this experiment is to determine the following two parameters of the proposed algorithm: the time length of the symbol *T* and the projection direction number of PLS regression *k*. To do this, three versions of datasets are generated with different symbol time length: T=0.3, 0.6 and 1 s. Next, the proposed method is evaluated with different projection direction numbers. 3 randomly-selected sessions are used for training and the rests for test. The final experiment results are computed by averaging the results of three independent executions.

**Figure 9:**
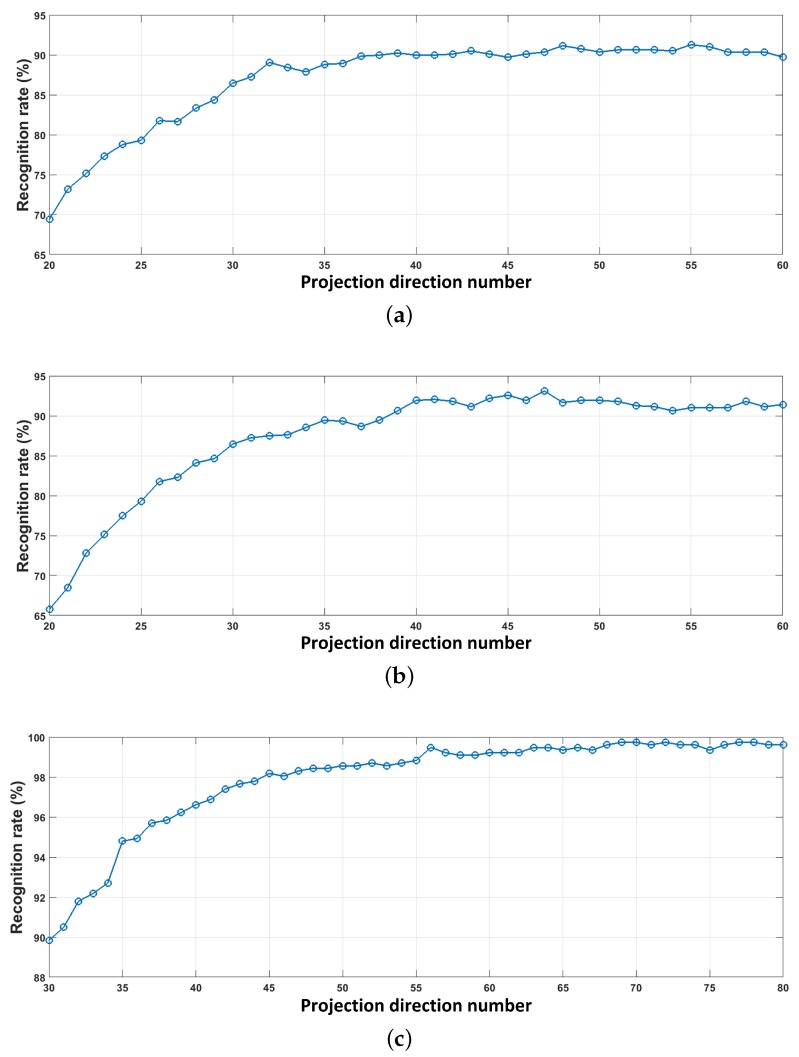
Recognition rate over the number of projection directions: SNR = −8 dB and fs=4000 Hz. (**a**) T=0.3 s; (**b**) T=0.6 s; (**c**) T=1 s.

We seek the optimal value of *k* by maximizing the recognition rate of the systems experimentally. The recognition rate is defined as the ratio of the correct classification counts and the number of input test symbols. This criterion has been widely used to evaluate the classification performance in pattern recognition field. Figure 9 plots the recognition rate curves of the three dataset versions over *k*. As expected, the recognition rate raises with the increasing of the *k* linearly at the beginning then slows down. It can be seen that the curves of T=0.3 and T=0.6 trend towards to stability when k=40, whereas T=1 when k=65. Meanwhile, the optimal recognition rates are obtained when *k* is 42, 55 and 70 for the three dataset versions, respectively. That is, the optimal recognition rates are 90.36%, 92.58% and 99.74% when *T* is equal to 0.3, 0.6 and 1 s with SNR=−8 dB.

Additionally, it is found also that the dataset with larger symbol time length leads to better accuracy. This is because the larger *T* is, the more time gains can be achieved in the slow-time-varying environment. This experiment demonstrates that the symbols with the period of 1 s lead to an accuracy of 99.74%, to nearly 100%, in a low signal-to-noise ratio environment (SNR=−8 dB).

### 5.3. Demodulation Accuracy Evaluation

The experiment of this subsection evaluates the accuracy performance of the proposed method by comparing it with two reference algorithms, including traditional matched filter [7] and a three-layer back propagation (BP) neural network [56]. Bit error rate is used as the criterion of accuracy performance. Both of the reference algorithms can be directly inserted into the scheme described in Section 3.1.

We compare the three demodulators with all of the three versions of the datasets: three of the six sessions for training, and the rests for testing. That is, for each data version, either the training or testing dataset has 256 symbol candidates × three sessions = 768 simulated symbols. For the purpose of unbiased evaluation, the simulated symbols are randomly divided into two groups. The average bit error rate of three repetitions with different training and testing data is used as the evaluation criterion of the demodulation accuracy performance.

**Figure 10:**
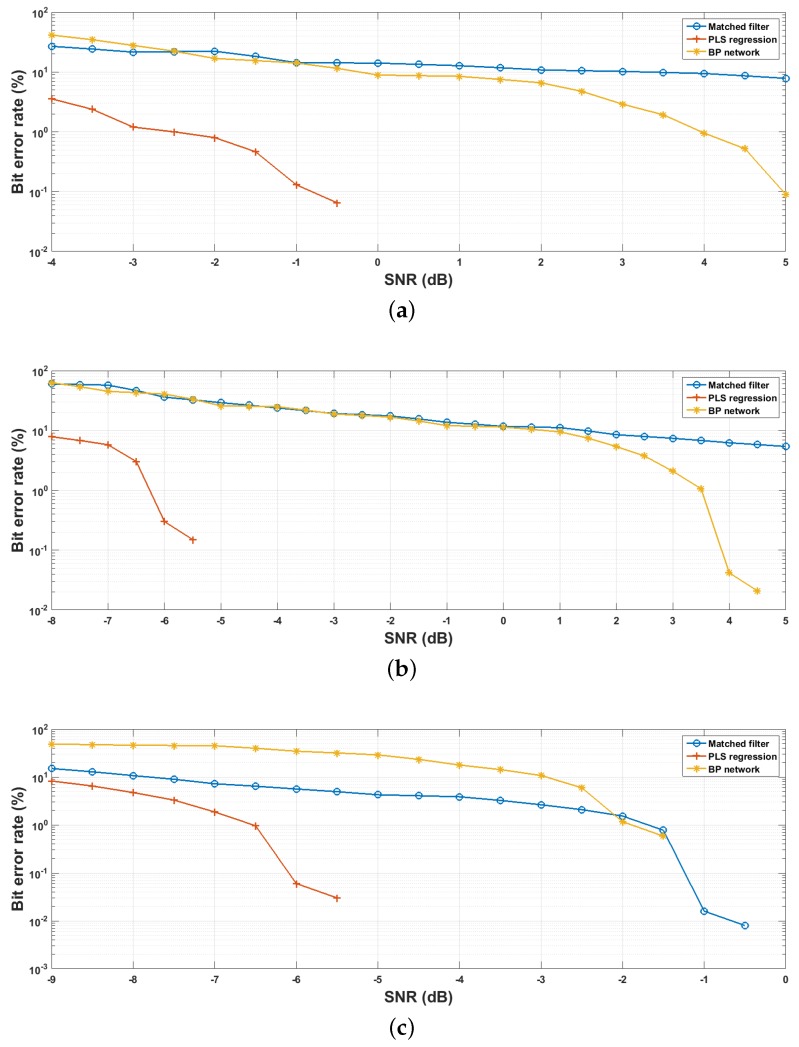
Accuracy comparison results with bit error ratios: fs=4000 Hz. (**a**) T=0.3 s, k=40; (**b**) T=0.6 s, k=55; (**c**) T=1 s, k=70.

Figure 10 compares the accuracy performance between the proposed method and the reference algorithms with the three versions of datasets. First of all, we can see that PLS regression always possesses the lowest bit error rates. For the three dataset versions, when SNR is around −2, −6 and −6.5 dB, the BERs of PLS reach 1.0%, whereas the matched filter and BP network are 22.24%, 36.05%, 6.44%, and 16.79%, 40.33%, 39.87%, respectively. Additionally, PLS achieves an error-free demodulation results with the three datasets at SNR=0,−5and−5 dB, out of the available range of the y-axis with logarithmic scale. This demonstrates that the proposed method has the best accuracy performance with the experiment protocol of this paper.

**Figure 11:**
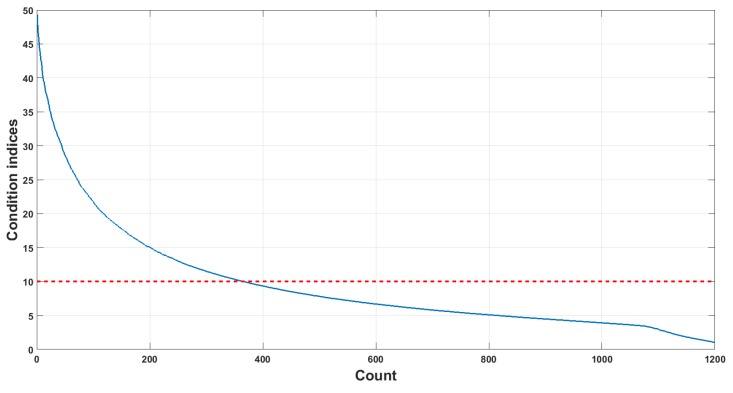
Condition indices of the 0.3-s symbol set.

In our case, the communication symbols are modulated with multi-segment piecewise linear mapping, which are essentially a set of pseudo-random sequences, implying that the predictors (elements of the symbol vector) are somehow correlated, resulting in a multicollinearity problem. The multicollinearity problem is a phenomenon where one predictor variable in a multiple regression model can be linearly predicted from the others with a substantial degree of accuracy, resulting in model distortions [57]. Mathematically, a set of variables is perfectly multicollinear if one or more exact linear relationships exist among some of the variables:

(22) $$\beta_{0}+\beta_{1}x_{1}+\beta_{2}x_{2}+\cdots +\beta_{i}x_{i}=0$$

where $\beta_{i}$ are constants and $x_{i}$ is the *i*-th element of the symbol vector $u_{n}^{\prime }$. We quantify the multicollinearity of the simulated dataset by using condition indices (CIs) [58]:

(23) $$CI_{i}=\sqrt{\frac{\lambda_{max}}{\lambda_{i}}}$$

where $\lambda_{max}$ is the maximum eigenvalue of the symbol set, and $\lambda_{i}$ is its *i*-th eigenvalue. Belsey et al. [59] suggest that when the value of CI is higher than 10, data dependencies are starting to affect the regression estimates. Figure 11 plots the condition indices of a 0.3-s symbol set, in which 30.42% of the elements are collinear with the others.

Unlike the reference algorithms, PLS models the fundamental relations between the training and response matrices by modeling their covariance structure. More precisely, a PLS model will try to find the multidimensional direction in the space of training matrix that explains the maximum multidimensional variance direction in the space of response matrix. The PLS family of methods is, therefore, particular suited for multicollinearity problem, providing better accuracy performance than the others.

As for the BP networks, the matched filters achieve better accuracy than when the SNR is low. In our opinion, this is caused by the fact that the power of the noise components is higher than the transmitted signals within low SNR environment (SNR<0 dB), whereas the given training examples are too sparse, resulting that the BP networks are drowned by the strong ambient noise and can hardly capture the useful feature information even if they are well converged. Yet, if the signal-to-noise ratio exceeds certain thresholds (in the case of this evaluation they are −1, 0 and −2 dB for the three datasets), the networks will provide better accuracy than matched filter. Additionally, the PLS models are trained by using the same datasets with the BP networks; it demonstrates, therefore, that incorporating variable relationship analysis into the regression problem can improve the capacity of the regressors in terms of handling the sparse training datasets.

Finally, the accuracy performance of the matched filters significantly rises with the increasing of the symbol time length *T*. For example, the bit error rates of the matched filters are around 14%, 11% and 0% at SNR=0 dB. As mentioned in Section 5.2, the dinsguishability of the symbols varies in function of its length due to the time gain. According to our further test, with the same experimental protocol, both matched filter and PLS regression achieve the 0% BER at SNR=−5 dB when T=1.5 s. However, it should be noted that the communication rate is inversely proportional to *T*, so the method of this paper is more appropriate when requiring a high communication rate.

### 5.4. Practicability Analysis

Collecting high-density qualitied underwater acoustic data is technically difficult for today’s acoustic engineering. In this subsection, the environment and sample quality compatibility is evaluated to investigate whether the training datasets could be sparse to a certain extent. The evaluations are performed by testing the well-trained PLS models using the datasets with strange underwater sound channels and different SNRs. To do this, we have 50% of the testing symbols simulated via strange channels (not included in the training channels). The time interval between the two channel measurements is around 5 minutes, and their normalized correlation is around 0.8. The evaluation result is shown in Table 2, in which the rows correspond to the training datasets and the columns to the testing datasets.

Comparing the diagonal of Table 2 with the simulation results of Figure 10b which trains and tests with the same sound channel, we can see that a strange channel somehow reduces the accuracy performance of the demodulator, but this impact is tiny, and the demodulator reaches the BER of 0% at SNR=−4 dB, which is still much better than either BP networks or matched filters. This demonstrates that the proposed method has the ability to resist the channel variant. Meanwhile, both of the SNRs of the training and testing datasets positively affect the accuracy of the demodulator. Poor training data do not necessarily result in high BERs, and vice versa. Hence, the performance of our demodulator is not seriously affected by the gap between the training and testing datasets, implying that the training data can be collected with few constraints.

### 5.5. Temporal Efficiency Evaluation

This subsection evaluates the temporal efficiency performance of the proposed demodulator by comparing it with the matched filter and BP network. This paper theoretically analyzes the efficiency performance of all the algorithms with asymptotic time complexity (also known as time complexity) [60]:

(24) T(n)=O(f(n))=ηf(n)

where n is the scale of the problem to be solved and f(n) is a function having the same order of magnitude with T(n), which makes their ratio η is a non-zero constant.

The pseudocode of the demodulation scheme shown in Figure 3 is given in Algorithm 2, in which Line 7 is replaced by using the matched filter or BP network to perform the comparisons. The time complexity of this demodulation scheme $T_{total}$ can be expressed as:

(25) $$T_{total}=T_{FIR}+T_{norm}+T_{*}+T_{Dec}$$

with

(26) $$\begin{array}{c} T_{FIR}=m\times T\times f_{s}\times n_{ord} \end{array}$$

(27) $$\begin{array}{c} T_{norm}=m\times T\times f_{s} \end{array}$$

(28) $$\begin{array}{c} T_{Dec}=\frac{3}{2}\times m\times n \end{array}$$

where $T_{FIR}$, $T_{norm}$ and $T_{Dec}$ are the time complexities of FIR filter, normalization operation and decision function, $T_{*}$ with ∗= “PLS”, “MF” or “BP” is the time complexity of the selected demodulator, $n_{ord}$ is the order of the FIR filter, *m* is the symbol number of the input message, n=256 is the number of symbol candidates, and $f_{s}=4000$ Hz is the sampling rate.

For each symbol, according to the algorithms presented in Section 3, the PLS model runs in polynomial time (An algorithm is said to be of polynomial time if its running time is upper-bound by a polynomial expression in the size of the input for the algorithm, i.e., $mathcalT\left(\approx ⤺\approx ℧n\right)=O\left(\approx ⤺\approx ℧n^{\kappa }\right)$ for some positive constant κ [60,61].): $T_{PLS}=n\times T\times f_{s}$. Similarly, the time complexity of the matched filter is $T_{MF}=n\times T^{2}\times f_{s}^{2}$. As for the forward propagation of a BP network, when the size of a hidden layer is n−1, its time complexity is $T_{BP}=\left(n-1\right)\times T\times f_{s}+n^{2}$. With the experiment protocol of this paper, PLS model clearly has the lowest time complexity, whereas matched filter the highest.

Table 3 lists the implementations of the proposed and the two reference algorithms. They are implemented by using the multi-core CPU platform presented in Section 4. The indices “*_ori*” and “*_opt*” refer to the original and optimized implementations, respectively. The detection step of the matched filter (see Equation (2)) and the message generation steps of all the three algorithms are parallelized. The running speed measurements cover all the accelerated steps, including detection and decision. The CPU used is Intel(R) Corel(TM) i7-6700 CPU 3.4 GHz. The ratio of the running time, known as acceleration ratio, is used as the efficiency evaluation criterion, and the running time of *mf_ori* with T=0.3 s (72.8 ms per symbol) is set as the measuring basis.

**Figure 12:**
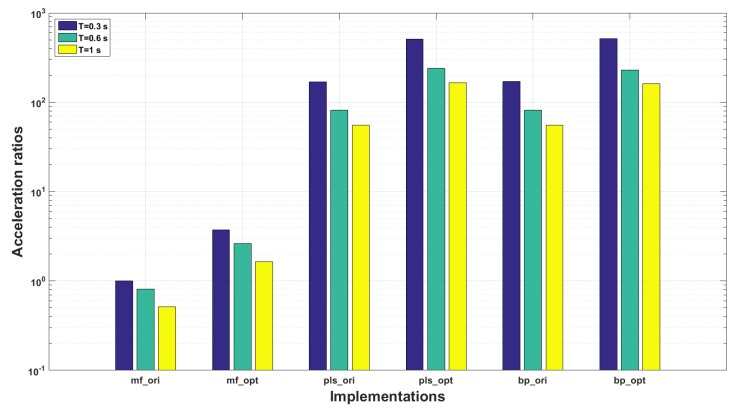
Running speed comparison results: $f_{s}=4000$ Hz.

Figure 12 displays the acceleration ratios of all the implementations. Firstly, we can see that the implementations based on machine learning methods are much more efficient than the matched filters. Their running speed gaps are hundreds of times. With matched filters, the received symbol is correlated with the replicas of the original symbols point by point along the time axis, which is quite time-costly. By contrast, the PLS model and the forward propagation process of BP networks are essentially matrix/vector multiplication operations with low computation intensity. So, even if the running speed has been multiplied through multi-thread optimization, their temporal efficiency performance are still not on the same order of magnitude.

Secondly, comparing with the original implementations, the optimized implementations are accelerated by around three times. In this experiment, a four-core CPU is used, so the speed-up should have been four times higher than measured values (in theory). This is caused by the following two reasons:

a the implementations are partly optimized instead of completely parallelized. The multi-thread optimizations of *pls_cpu* and *bp_cpu* cover only the message generations without detection steps;
b the shared memory architecture is constrained by the von Nuemann bottle neck, the memory access conflicts may be occurred when multiple thread functions are invoked simultaneously.

Depending on the efficiency evaluation result, the time costs of all the implementations are lower than *T*, satisfying the constraints of real-time processing. Therefore, the communication rate R is only constrained by *T*. Given that a symbol represents a bit stream of 8-bits with ASCII code, R can be estimated as $\left(8\times m\right)/\left(m\times T+d/c_{s}\right)$ in theory, where *m* is the received symbol number or the size of the transmitted message, *d* is the distance between the sender and receiver and $c_{s}$ is the underwater sound speed.

## 6. Conclusions

This paper presents a novel machine learning available demodulation scheme for underwater communication applications based on the chaos phase modulation spread spectrum techniques. Thanks to the characteristic of a large number of phase-shift keying, it is more appropriate for confidential communication applications. Compared to the reference algorithms, experiments demonstrate that the proposed approach possesses higher accuracy performance with low SNR and high communication rate. The main originalities and contributions of this paper include:

- The proposed demodulation scheme handles the signal demodulation as a target recognition problem, allowing us to incorporate advanced classification methods into it, benefiting from the recent progresses of pattern recognition techniques. Because machine learning methods are able to statistically analyze the symbol features and noise characters, it can provide better demodulation accuracy performance than matched filters with shorter symbol sequences. That is, if the same accuracy performance is desired, the conventional matched filters need long symbol sequences in order to get enough time gains, so adopting machine learning methods, especially PLS regression, can considerably improve the underwater communication applications in terms of communication rates;
- It is found that the chaos-spreading spectrum signals have the characteristic of multicollinearity which may potentially impact the performance of certain classifiers negatively. Fortunately, the algorithm family of PLS can somehow overcome this disadvantage by modeling the relationships between the predictor and response variables, even with a sparse training dataset;
- The running cost of the proposed approach is very low. The detection steps of the proposed scheme costs only several milliseconds with the experiment protocol of this paper, which even can be ignored during the overall demodulation procedure, so it possesses high temporal efficiency performance;
- The proposed demodulation algorithm can be easily transplanted to other hardware platforms for different purposes. The detection process of the proposed algorithm is actually an operation of matrix production, which can be easily implemented and optimized by using any currently available computing platforms.

Meanwhile, some issues exist still. Firstly, the spatial variations and frequency-shift characteristic of underwater sound propagations are not considered in the simulation; some more evaluations with real-life experiment data are strongly required in future studies. Secondly, the experiment of this paper demonstrates that PLS regression has better accuracy performance than BP networks. However, it cannot be concluded that PLS regression is always the best classifier for all the three mentioned methods, because the training set of this paper is sparse, far away from the requirement of deep learning. In the future work, we aim to attempt to further improve the evaluation experiment with the standard of deep learning. Thirdly, the PLS-based demodulator is able to resist some variation of communication channel, but it should be noted that the underwater sound channels sometimes rapidly vary in real-life applications. The training frequency of the PLS model will, therefore, be studied and a solution with real-time learning will be investigated. Finally, the parallel implementation of the training process of the proposed algorithm is not discussed in this paper. Some optimization properties within the instruction and data levels with other refined development tools, i.e., MPI for multi-core CPUs or CUDA for GPUs, still exist and are worth being investigated further.

## Figures and Tables

**Table 1:** Experimental parameters of underwater sound channel estimation.

Parameters	Values	Descriptions
Sea depth	90–100 m	Minimum and maximum sea depths during the propagation.
Distance	16.5 km	Both of the sender and receiver are stationary during the transmission.
Source level	200 dB	-
Impulse period	2 s	-
Impulse form	Chaotic	-
Sampling rate $f_{s}$	4000 Hz	-
Channel tap number $W_{h}$	400	$W_{h}=f_{s}\times 0.1$ s.
Channel estimation interval	10 ms	-

**Table 2:** BER (%) between the training and testing datasets with different underwater sound channel response and SNRs (dB): T=0.6 s, k=55 and $f_{s}=4000$ Hz, the rows correspond to the training datasets and the columns to the testing datasets.

		SNR	−10	−9	−8	−7	−6	−5	−4	−3	−2	−1	0
	BER												
SNR													
−10			44.61	33.57	22.77	21.21	13.78	8.98	6.91	4.52	4.52	3.98	2.76
−9			30.78	25.09	10.32	9.87	6.31	5.92	3.33	2.51	0,91	0.69	0.54
−8			27.14	14.12	9.75	5.01	3.82	2.01	1.21	0.82	0.51	0.42	0.21
−7			15.34	7.82	4.98	4.57	1.27	0.98	0.39	0.26	0.13	0.00	0.00
−6			10.52	4.21	2.61	1.55	0.26	0.13	0.00	0.00	0.00	0.00	0.00
−5			6.45	3.13	1.27	0.52	0.26	0.13	0.00	0.00	0.00	0.00	0.00
−4			4.33	1.01	0.13	0.13	0.13	0.00	0.00	0.00	0.00	0.00	0.00
−3			3.78	0.26	0.13	0.00	0.00	0.00	0.00	0.00	0.00	0.00	0.00
−2			2.21	0.51	0.00	0.00	0.00	0.00	0.00	0.00	0.00	0.00	0.00
−1			1.88	0.31	0.00	0.00	0.00	0.00	0.00	0.00	0.00	0.00	0.00
0			0.91	0.26	0.00	0.00	0.00	0.00	0.00	0.00	0.00	0.00	0.00

**Table 3:** Description of multi-core CPU implementations.

Names	Algorithms	Optimzations
*mf_ori*	Matched filter	None
*mf_opt*		Multi-thread parallelization
*bp_ori*	BP network	None
*bp_opt*		Multi-thread parallelization
*pls_ori*	PLS regression	None
*pls_opt*		Multi-thread parallelization
